# Dehydrin-like Proteins in the Necrotrophic Fungus *Alternaria brassicicola* Have a Role in Plant Pathogenesis and Stress Response

**DOI:** 10.1371/journal.pone.0075143

**Published:** 2013-10-02

**Authors:** Stéphanie Pochon, Philippe Simoneau, Sandrine Pigné, Samuel Balidas, Nelly Bataillé-Simoneau, Claire Campion, Emmanuel Jaspard, Benoît Calmes, Bruno Hamon, Romain Berruyer, Marjorie Juchaux, Thomas Guillemette

**Affiliations:** 1 Université d’Angers, UMR 1345 IRHS, SFR QUASAV, Angers, France; 2 INRA, UMR 1345 IRHS, Angers, France; 3 Agrocampus-Ouest, UMR 1345 IRHS, Angers, France; 4 Université d’Angers, SFR QUASAV, IMAC, Beaucouzé, France; Geisel School of Medicine at Dartmouth, United States of America

## Abstract

In this study, the roles of fungal dehydrin-like proteins in pathogenicity and protection against environmental stresses were investigated in the necrotrophic seed-borne fungus *Alternaria brassicicola*. Three proteins (called AbDhn1, AbDhn2 and AbDhn3), harbouring the asparagine-proline-arginine (DPR) signature pattern and sharing the characteristic features of fungal dehydrin-like proteins, were identified in the *A. brassicicola* genome. The expression of these genes was induced in response to various stresses and found to be regulated by the AbHog1 mitogen-activated protein kinase (MAPK) pathway. A knock-out approach showed that dehydrin-like proteins have an impact mainly on oxidative stress tolerance and on conidial survival upon exposure to high and freezing temperatures. The subcellular localization revealed that AbDhn1 and AbDhn2 were associated with peroxisomes, which is consistent with a possible perturbation of protective mechanisms to counteract oxidative stress and maintain the redox balance in AbDhn mutants. Finally, we show that the double deletion mutant *ΔΔabdhn1-abdhn2* was highly compromised in its pathogenicity. By comparison to the wild-type, this mutant exhibited lower aggressiveness on *B. oleracea* leaves and a reduced capacity to be transmitted to *Arabidopsis* seeds via siliques. The double mutant was also affected with respect to conidiation, another crucial step in the epidemiology of the disease.

## Introduction

Dehydrins belong to the 2a and 2b groups of the large late embryogenesis-abundant (LEA) protein family [1], [2], which were initially described as accumulating late in plant seed development. They were also found in vegetative plant tissues following environmental stresses and are believed to play a role in the protection against cold- and dehydration-related stresses [3], [4], [5]. The dehydrin family seems to be widely distributed as homologous sequences were found in invertebrate and microorganism genomic sequences [6]. Besides the sequence similarities, dehydrins share typical physicochemical features. Their amino acid composition is thus characterized by high percentages of glycine, threonine and serine, and low levels of cysteine and tryptophan residues. Moreover, they exhibit high hydrophilicity, an absence of secondary structures, a high proportion of disordered amino acids and can thus be considered as intrinsically unstructured proteins (IUPs). Disordered regions in dehydrins may constitute flexible linkers or spacers that have a role in forming macromolecular assemblies. In virtue of this structural plasticity, IUPs may serve as potent chaperones [7]. Moreover, a distinctive feature of plant group 2 LEA proteins is a conserved Lys-rich motif named K-segment [8] usually found in the N terminus of the protein. The K-segment motifs are predicted to form amphipathic α-helical structures similar to the lipid binding class A2 amphipathic α-helical region found in apolipoproteins associated with membranes [9]. This observation raised the hypothesis that one of the roles of the plant dehydrins may be related to membrane stabilization during stress through interaction with hydrophobic surfaces [10].

In fungi, dehydrin-encoding genes have been identified in *Tuber borchii* and *Aspergillus fumigatus* during searches for genes controlling fruiting body maturation or conidial dormancy [6], [11]. Recently, Wartenberg et al [12] also identified a farnesol-induced dehydrin-like protein (DlpA) in *A. nidulans*. Homologs were found in ascomycetous fungi but not in other fungal lineages. Their amino acid sequences harbor a repeated and conserved asparagine-proline-arginine (DPR) motif, corresponding to a fungal dehydrin signature pattern [6], while a relatively poor sequence homology is generally found outside of DPR motifs. The corresponding transcripts generally accumulate in the same conditions as those affecting plant DHNs transcription, e.g. in response to hyperosmosis, low temperature and salinity stresses. The three dehydrin-like proteins (DprA, DprB and DprC) identified in *A. fumigatus* and DlpA in *A. nidulans* were also up-regulated upon addition of dithiothreitol (DTT), an inducer of protein misfolding, and were described as stress protective molecules [11], [12], [13]. They notably contribute to protection against high pH, freezing, osmotic and oxidative stress. According to these features, they were presumed to function as molecular chaperones or membrane stabilizers. Different locations have been assigned to them. While DprC was associated with the vacuoles, DprA and DprB accumulated in the cytoplasm and peroxisomes. The authors hypothesized a peripheral association of DprA and DprB proteins with the peroxisome due to the absence of peroxisomal-targeting signal. Loss of DprA and DprB was not found to be associated with attenuated virulence in mice even though the conidia of mutants were hypersensitive to killing by lung phagocytes.

The necrotrophic fungus *Alternaria brassicicola* causes black spot disease and is an economically important seed-borne pathogen of Brassicaceae species. During host infection, *A. brassicicola* is exposed to high levels of defence compounds, such as phytoalexins and glucosinolate breakdown products, and the ability to overcome these antimicrobial metabolites is a key factor in determining fungal virulence. Seed transmission is also a major component of pathogen fitness, as *A. brassicicola* strongly depends on this process for its dispersal and long-term survival [14]. In this study, we identified three dehydrin-encoding genes (called *AbDhn1*, *AbDhn2* and *AbDhn3*) in the *A. brassicicola* genome via their sequence similarities and physicochemical characteristics. Expression analyses indicated that dehydrin gene transcription was dependent on the AbHog1 MAP kinase and that two AbDHN2 isoforms could be produced through alternative splicing of mRNA. We then investigated their role in protection against dehydration-related stresses and, for the first time, we analysed their impact on the pathogenicity during host vegetative tissue infection or the seed transmission process.

## Results

### AbDhn1, AbDhn2 and AbDhn3 Encode Three Dehydrin-like Proteins

**Figure 1:**
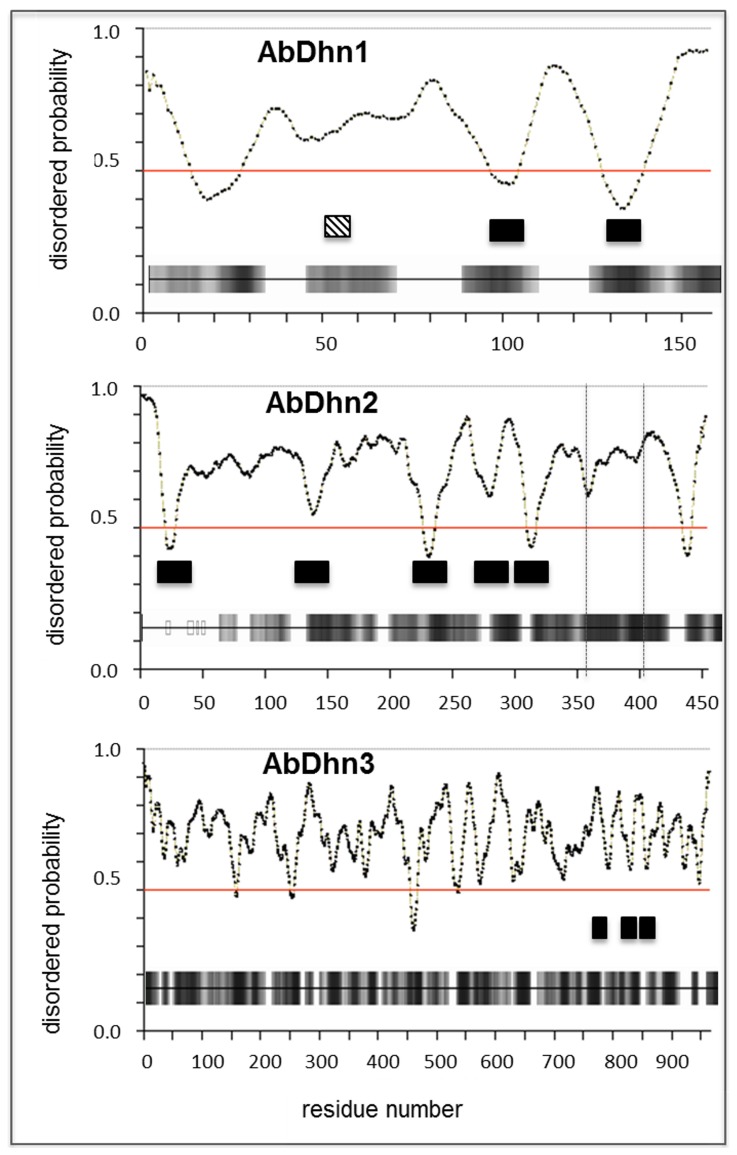
Disorder profiles of the three dehydrin-like proteins from *A. brassicicola*. Disorder probabilities are plotted according to the residue positions. Residues beyond the red threshold line in these plots are predicted to be disordered. Location of DPR domains (black box) and a predicted TRG_PEX motif (hatched box) are indicated. Predicted binding regions are shown below plots by horizontal bars shaded according to the prediction score. Residues between the vertical dotted lines on the AbDhn2 plot are missing in the ß isoform.

**Figure 2:**
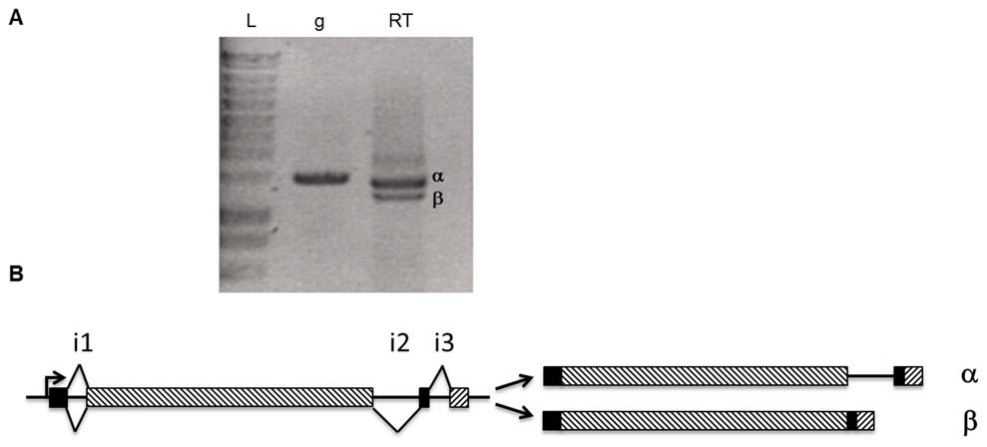
Alternative splicing of the *AbDhn2* transcript. A: Electrophoresis gel of PCR products obtained after amplification of the *AbDhn2* coding sequence using genomic DNA (lane g) or first-strand cDNA (lane RT) as template; Lane L: DNA ladder. B: Schematic representation of the splicing events leading to α and β forms of mature transcripts. Exons are indicated as black or hatched boxes.

The camalexin-induced sequence P3E9 (GenBank accession No DY543081) was previously shown to encode a 157-residue protein that exhibits similarities to dehydrin-like proteins from *Tuber borchii* [15]. This protein, here referred to as AbDhn1, indeed contained two amino acid repeats (**Fig. 1**) that corresponded to the signature pattern of fungal dehydrins [6] recently named DPR domains [11]. A BLAST search was conducted in the *A. brassicicola* automatically annotated genome database (http://genome.jgi-psf.org/Altbr1) to check for proteins containing similar motifs. Besides a protein referred to as AB02513.1 corresponding to AbDhn1, two other hits (AB08993.1 and AB05365.1) were found and named AbDhn2 and AbDhn3 (GenBank accession No JX891381 and JX891382), respectively. The nucleotide sequence encoding the latter protein was located at the end of a contig and 3′ RACE was performed to obtain the C-terminal encoding sequence. The complete AbDhn3 protein contains 964 amino acids and three DPR motifs (**Fig. 1**). The automatic annotation at the locus encoding AB08993.1 predicted four introns. Amplification of first-strand cDNA with primers spanning the whole coding sequence generated two products differing of ca 150 bp in size (**Fig. 2A**). Analysis of the nucleotide sequences of the two amplicons and comparison with the corresponding genomic sequence suggested that the *AbDhn2* gene contains two conventional 54 bp- and 57 bp-introns near the 5′ and 3′ ends respectively, both being absent in the amplified cDNA sequences, and a third longer 159 bp-intron only present in the larger amplification product (**Fig. 2B**). Blast search in the *A. brassicicola* genome assembly database and Southern hybridization (**Fig. S1**) did not reveal any additional AbDhn2 coding sequence, strongly suggesting that the two transcripts were produced by alternative splicing of a *AbDhn2* pre-mRNA. The two deduced AbDhn2 protein isoforms contain 453 amino acids (α isoform) and 400 amino acids (β isoform) and five DPR motifs (**Fig. 1**). A stretch of eleven amino acids repeated seven times was also observed at the N-terminal part of their sequence (**Fig. S2**).

**Figure 3:**
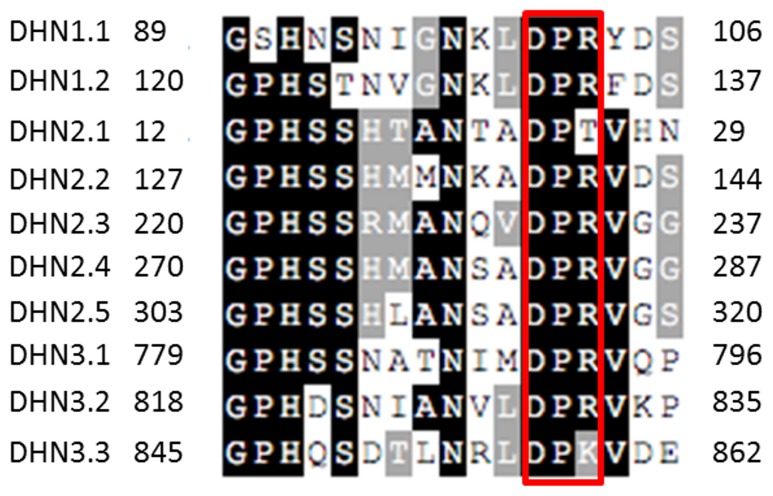
Alignment of the repeated DPR domains of AbDhn1, AbDhn2 and AbDhn3. Conserved amino acids are boxed in black (identical) or grey (similar). DHN1.1–DHN1.2 designate the two domains from AbDhn1, DHN2.1–DHN2.5 the five domains of AbDhn2, and DHN3.1–DHN3.3 the three domains from AbDhn3. Numbers indicate the amino acid positions. The conserved DPR motif is boxed in red.

The alignment of the DPR domains from the three *A. brassicicola* dehydrin-like proteins is shown in **Fig. 3**. Apart from the conserved domains, sequence comparison of these proteins revealed very poor homology, and sequences producing significant alignments (>50% identity and coverage) were only detected in genomes of closely related Pleosporales species (*A. arborescens, Pyrenophora tritici-repentis, P. teres, Phaeosphaeria nodorum*). However they all shared typical features of dehydrins such as high glycine, threonine and serine content, low cysteine and tryptophan content, high hydrophilicity, i.e. negative GRAVY values (**Table 1**). Moreover they lack well-defined tertiary structure as revealed by their high percentage of amino acids predicted as being disordered and as potential binding regions (**Fig. 1**). In AbDhn1, one of these potential binding regions contained a Wxxx[YF] motif found in proteins involved in peroxisomal matrix import into peroxisomes.

**Table 1:** Characteristics of the dehydrin-like proteins found in *A. brassicicola.*

	Gly*	Ser*	Thr*	Cys*	Trp*	Gravy value
**AbDHN1**	14	7	10	0	0.6	−1.053
**AbDHN2**α	21	13	16	0	0	−0.879
**AbDHN2β**	22.5	13.7	17.7	0.1	0	−0.855
**AbDHN3**	10.3	10.3	9.5	0	0	−0.827

* values indicate percentage of total amino acids.

### Stress-regulated Expression of AbDhn Genes

**Figure 4:**
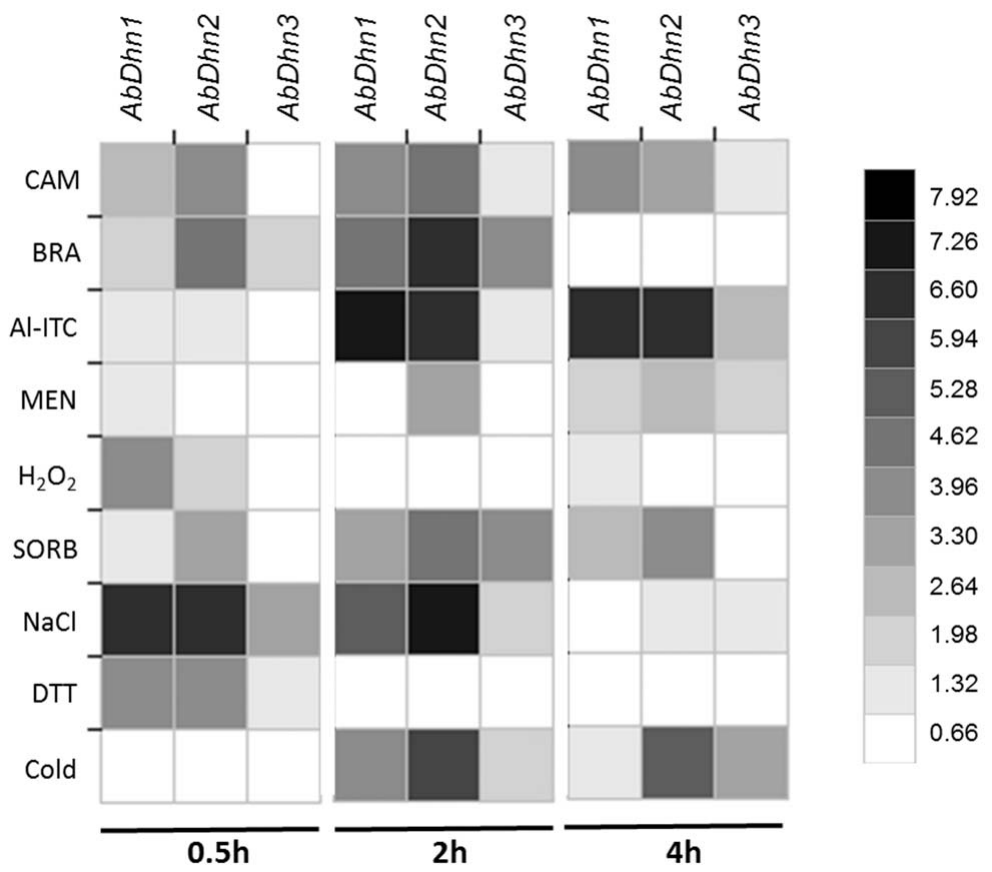
Expression levels of *AbDhn1*, *AbDhn2* and *AdDhn3* sequences in *A. brassicicola* exposed to various stresses. First-strand cDNAs were prepared from RNA samples extracted from germinated conidia either exposed to 125 µM camalexin (CAM), 125 µM brassinin (BRA), 2.5 mM allyl-isothiocyanate (Al-ITC), 5 mM menadione (MEN), 5 mM hydrogen peroxide (H2O2), 1 M sorbitol (SORB), 350 mM sodium chloride (NACL), 20 mM dithiothreitol (DTT) or incubated at 4°C (COLD) for the indicated times and used as template for real-time PCR. For each gene, expression induction is represented as a ratio (fold induction) of its relative expression (studied gene transcript abundance/β-tubulin transcript abundance) in each inductive condition to its relative expression in the corresponding control. Each value is the mean of two independent experiments, each with three replicates. For easier visualization of the results, numerical data were transformed into colour-grid representations using JColorGrid software [45] in which the fold gene expression induction (Log_2_ values) is represented by a grey scale (on the right).

Previous experiments showed that the *AbDhn1* gene was upregulated during exposure of *A. brassicicola* to the indolic phytoalexin camalexin [16]. We thus investigated the expression of the three dehydrin-like genes in germinated conidia exposed to camalexin but also to other brassicaceous defense metabolites, i.e. the indolic phytoalexin brassinin, and allyl-isothiocyanate (Al-ITC), a breakdown product of the aliphatic glucosinate sinigrin. As shown in **Fig. 4**, *AbDhn1* and *AbDhn2* transcription levels increased right after 0.5 h of exposure to camalexin and brassinin and the highest transcripts levels were observed after 2 h of treatment with the two phytoalexins. Increased *AbDhn1* and *AbDhn2* expressions were also observed in germinated conidia following 2–4 h of exposure to Al-ITC.

Transcript levels were also measured for the three dehydrin-like genes in conditions previously reported to induce fungal DHN transcription, i.e. low temperature-, salinity-, osmotic- and oxidative-stress [6], [11], [12], [13]. *AbDhn1* and *AbDhn2* transcript levels dramatically increased immediately following transfer on NaCl containing medium for 0.5 h and up to 2 h, while this upregulation was no longer observed after prolonged (4 h) treatment. A similar strong upregulation response was observed for *AbDhn2* after 2–4 h of exposure to cold temperature. Increased expression was also observed for *AbDhn1* in the presence of H_2_O_2_ and for *AbDhn2* and *AbDhn3* in the presence of sorbitol, but at much lower levels.

**Figure 5:**
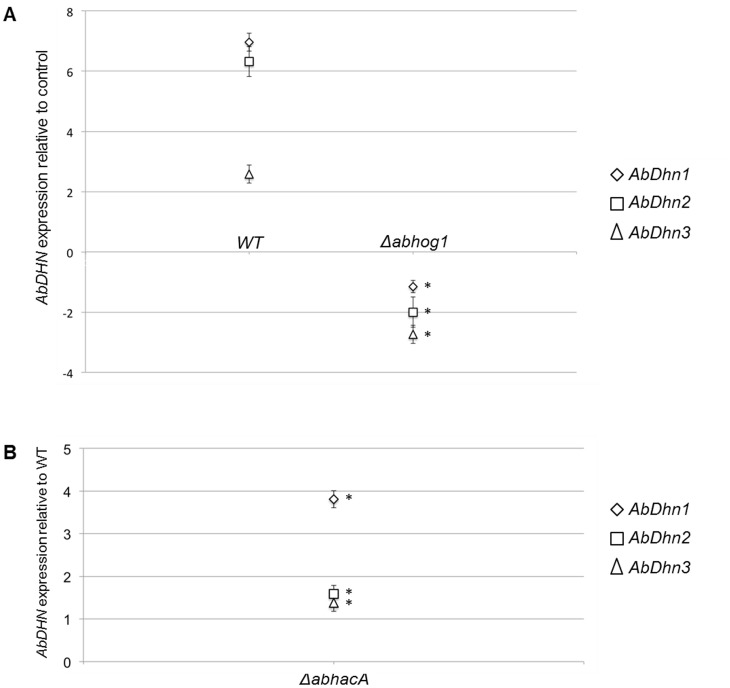
Expression of *AbDhn* genes in different genetic backgrounds estimated by real-time PCR. A: *A. brassicicola* wild-type (WT) and Δ*abhog1* strains were exposed to 350 mM NaCl for 30 min prior RNA extraction. For the three *AbDhn* genes, expression induction is represented as a log_2_ ratio (fold induction) of their relative expression under stress condition to their relative expression in the control without stress. B: Basal transcript levels of *AbDhn* genes in the Δ*abhacA* mutant relative to their expression levels in the reference wild-type strain *Abra 43* (Log_2_ values). Each value is the mean of two independent experiments, each with three replicates. Asterisks indicate values that are significantly (*P*<0.01) different than that of the wild-type.

In *A. brassicicola* the Hog1 MAPK cascade plays an important role in the response to camalexin exposure, oxidative stress [16] and salt stress (unpublished results). To check whether dehydrin-like genes constitute potential targets for this signalling cascade, their expression was assessed in a Δ*abhog1* mutant. As shown in **Fig. 5A**, significantly decreased accumulation of the *AbDhn* transcripts was observed in the MAPK-deficient strain compared to the wild-type under inducing conditions (NaCl stress). As camalexin has also been shown to activate the unfolded protein response (UPR) pathway in *A. brassicicola* [17], and due to the potential molecular chaperone function of intrinsically unstructured proteins (IUPs), expression of the three dehydrin-like genes was investigated, either in the wild-type strain exposed to the chemical UPR-inducer dithiothreitol (DTT), or in the UPR-defective Δ*abhacA* mutant without exogenous stress application. Upon treatment with DTT for 0.5 h, significant upregulation of *AbDhn1* and *AbDhn2* expression was observed in the wild-type strain (**Fig. 4**). These inductions were not observed for longer DTT exposure times. In the absence of exogenous stress, a dramatic increase in *AbDhn1* transcripts was observed in the Δ*abhacA* mutant, deficient for the transcription factor AbHacA, as compared to the wild-type (**Fig. 5B**).

### Dehydrin-like Proteins AbDhn1 and AbDhn2 Accumulate under Salt Stress in Peroxisomes

**Figure 6:**
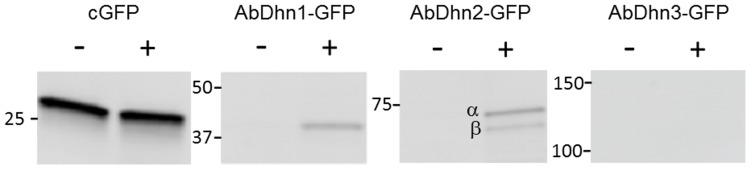
Expression of AbDhn-GFP proteins under salt stress. Germinated conidia (24 h-old) from strains expressing AbDhn-GFP fusions under the control of their own promoters or from a strain constitutively expressing GFP (cGFP) were either exposed to 350 mM NaCl for 2 h or left without stress. Proteins extracts were immunoblotted with HRP-coupled antibodies directed against GFP. Numbers correspond to molecular weights in kDa. The two AbDhn2 isoforms are indicated.

To check whether increased levels of dehydrin transcripts were paralleled by the accumulation of dehydrin-like proteins, strains expressing AbDhn proteins, under the control of their own promoters and fused at their carboxy-terminal end to sGFP, were engineered. As a control, a strain expressing only sGFP under control of the *ToxA* promoter [18] was also constructed. Proteins extracted from each strain grown in liquid cultures in the presence or absence of NaCl were analysed by Western blot using anti-GFP antibodies. As shown in **Fig. 6**, under basal growth conditions no signal was observed except with proteins extracted from the control strain constitutively expressing GFP. After salt-stress exposure, GFP antibodies recognized one protein at ca 40 kDa in extract from the strain expressing AbDhn1-GFP and two proteins at ca 65–70 kDa, i.e. that could correspond to the α and β isoforms of AbDhn2, in extracts from the strain expressing AbDhn2-GFP. No signal was observed with extracts from the strain expressing AbDhn3-GFP, suggesting that this protein was not expressed or at a very low level at least under the salt stress conditions.

**Figure 7:**
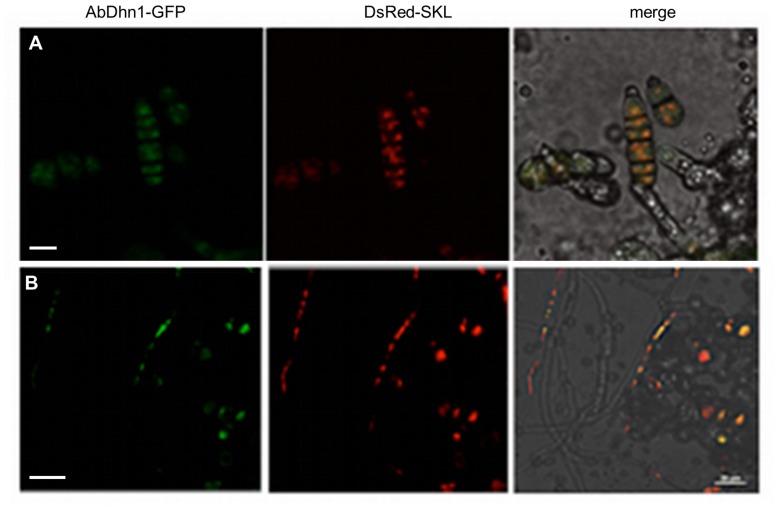
Subcellular localization of the AbDhn1-GFP fusion protein. Double-labelled strains expressing AbDhn1*-*GFP and DsRed-SKL were exposed to 350 mM NaCl for 2 h. Co-localization analyses in conidia (A) and hyphae (B) were examined using confocal microscopy. Bars = 10 µm.

The subcellular localization of AbDHN proteins was checked using the same strains. The GFP-Dhn1 signals formed punctate green dots in conidium cells (**Fig. 7A** **)** and young vegetative hyphae (**Fig. 7B**) that tended to be larger in older hyphae. Similar expression patterns were observed with the strain expressing AbDhn2-GFP, whereas GFP expression in the control strain resulted in diffuse green fluorescence (**Fig. S3**). Based on previously published results on dehydrin-like proteins from *A. fumigatus,* a peroxisomal localization of AbDhn1 and AbDhn2 proteins was hypothesised. To achieve labeling of peroxisomes, the red fluorescent protein DsRed was fused to a serine-lysine-leucine tag (SKL) typical of the type 1 peroxisomal targeting sequence PTS1. Expression of DsRed-SKL in *A. brassicicola* leads to the import of DsRed into peroxisomes and results in the detection of fluorescence signals after excitation. In the transformants that co-expressed AbDhn1- or AbDhn2-GFP and DsRed-SKL, co-localization of sGFP and DsRed was observed in conidia and hyphae (**Fig. 7**), confirming that the two dehydrin-like proteins were associated with peroxisomes. For Dhn3, only faint fluorescent signals were observed at the tip of hyphae but the cellular localization could not be accurately determined (not shown).

### Dehydrin-like Deficient Mutants are Susceptible to Oxidative Stress

**Figure 8:**
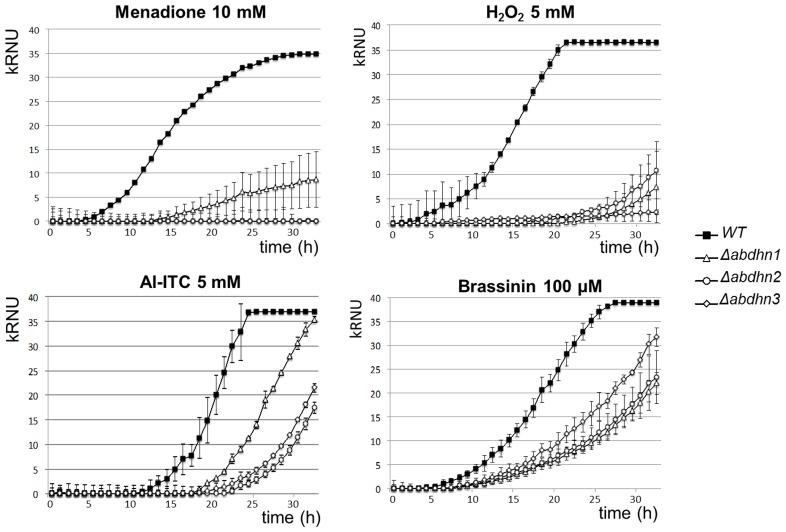
Susceptibility of AbDhn-deficient mutants to oxidative stress. Nephelometric monitoring of growth of wild-type strain (black symbols) and AbDhn-deficient mutants (open symbols; Δ*abdhn1*: triangles, Δ*abdhn2*: circles, Δ*abdh3*: diamonds) was automatically recorded for 33 h at 24°C. The unit of the Y-axis corresponds to the Relative Nephelometric Unit (RNU). Conidia were used to inoculate microplate wells containing standard PDB medium that was supplemented with either 10 mM menadione, 5 mM H_2_O_2_, 5 mM Al-ITC or 100 µM brassinin. Error bars indicate standard deviations. Each genotype was analysed in triplicate and the experiments were repeated twice times per growth condition. Lag time and maximal growth rate variables were calculated from the growth curves using a calculation method described by Joubert et al. [19]. For each parameter, Student’s T-test was used to assess significant difference between the treated and untreated samples or between mutants and parental isolate.

To investigate the role of dehydrin-like proteins in *A. brassicicola*, knockout mutants deficient for *AbDhn1, AbDhn2* or *AbDhn3* were constructed by replacing the respective ORFs with a hygromycin B-resistance cassette (**Fig. S1A**). The resulting replacement mutants (called Δ*abdhn1*, Δ*abdhn2*, and Δ*abdhn3*) were selected among hygromycin-resistant transformants using a PCR screen (not shown). For each genotype, disruption of *Dhn* genes was confirmed by DNA gel blot analysis (**Fig. S1B**) in two to three randomly selected transformants. Besides the expected signals, the DNA gel blot analysis did not detect any additional fragments, implying that there were no structural homologs of *AbDhn1, AbDhn2* or *AbDhn3* in *A. brassicicola*. Monitoring growth in solid PDA medium (**Fig. S4A**) and in liquid PDB medium (**Fig. S4B**) did not reveal any effect of single mutations compared to the wild-type parental strain on the mycelium growth rate, conidia germination and initial hyphal growth. Analyses of growth curves in liquid medium supplemented with menadione (generation of O_2_^−^), H_2_O_2_, Allyl-isothiocyanate (Al-ITC) and brassinin were used to assess the susceptibility of the dehydrin-like mutants to oxidative stress and plant defense metabolites. For a given genotype, all of the tested transformants had a similar response to the applied stress and mean growth curves were thus constructed. Lag time and maximal growth rate variables were calculated from the growth curves using a calculation method described by Joubert et al [19]. For each parameter, Student’s T-test was used to assess significant difference between the treated and untreated samples or between mutants and parental isolate. As shown in **Fig. 8**, all the dehydrin-like mutants were characterized by high susceptibility to oxidative stress. Almost complete growth inhibition of the mutant strains was observed in the presence of 10 mM menadione and 5 mM H_2_O_2_ while in the same conditions the growth of the wild-type was not significantly affected (**Fig. 8** **)**. Similarly, Al-ITC induced delayed entry into the log phase (ca. 7 h) and brassinin caused a significant reduction in the maximum slope (from 23% for Δ*abdhn3* to 42% for Δ*abdhn1*) for the three dehydrin-like deficient mutants compared to the wild-type (**Fig. 8**).

Similar experiments were performed in the presence of NaCl and sorbitol to assess the susceptibility of the mutant strains to salt- and osmotic-stress, respectively. None of the dehydrin-like deficient mutants showed increased susceptibility to any of these stresses compared to the wild-type (data not shown). As the expression patterns of *AbDhn1* and *AbDhn2* were quite similar, a possible functional redundancy was suspected and an *AbDhn1-AbDhn2* double deletion mutant was constructed by transforming a *Δabdhn1* mutant strain with an *AbDhn2*-replacement cassette containing a nourseothricin-resistance marker (**Fig. S1A**). The double gene replacement strains (called *ΔΔabdhn1-abdhn2*) were selected for both nourseothricin and hygromycin resistance. DNA gel blot analysis revealed that these transformants had lost both the *AbDhn1* and *AbDhn2* coding sequences that were replaced by the two disruption cassettes (**Fig. S1B**). In both liquid and solid media, growth retardation was observed as compared to other genotypes and the mycelial colony formed by the *ΔΔabdhn1-abdhn2* mutant was not darkly melanised (**Fig. S4**) due to weak conidia production. No other phenotypic difference was recorded between the double mutant and the *Δabdhn1* and *Δabdhn2* single mutant strains when grown in the presence of salt or sorbitol (data not shown).

### Conidia from Dehydrin-like Deficient Mutants Display Altered Survival Rates upon Exposure to High and Freezing Temperatures

**Figure 9:**
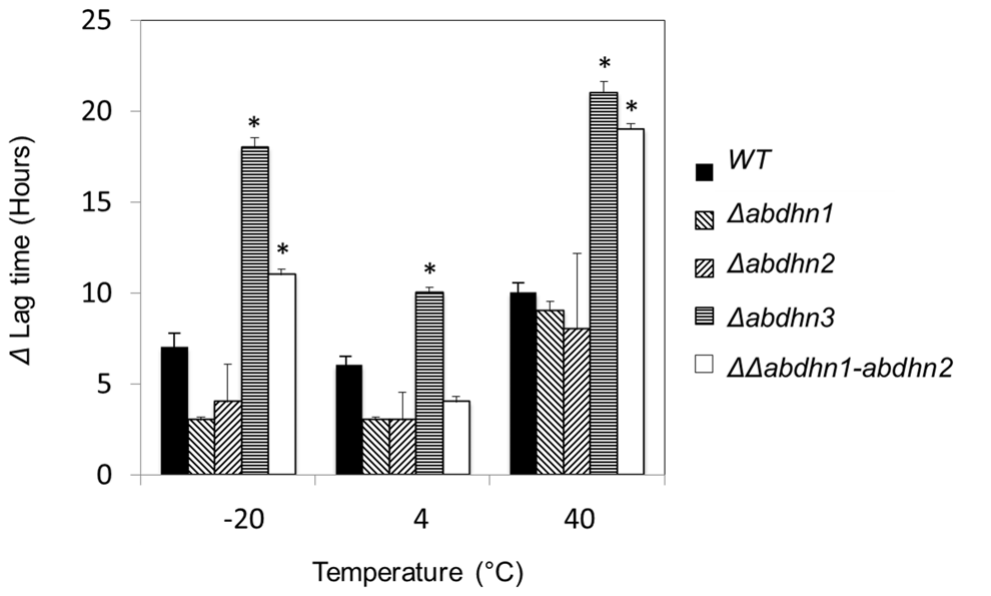
Susceptibility of AbDhn-deficient mutants to temperature stress. Calibrated water suspensions of conidia from the wild-type (WT) strain *Abra43* and AbDhn-deficient mutants were left for 10 h at various temperatures (−20°C, +4°C, +20°C, +40°C). Conidia were then used to inoculate microplate wells and nephelometric growth curves were established over a 33 h period. ΔLag time was calculated as the difference between the lag time at the tested temperature and the lag time at 20°C and was used as a parameter to estimate the effect of the treatment on spore viability. Error bars indicate standard deviations and asterisks indicate values that are significantly (*P*<0.01) higher than that of the wild-type. Each genotype was analysed in triplicate and the experiments were repeated twice times per growth condition.

Nephelometric recording of growth was used to calculate lag times and assess conidia germination after 10 h of storage in water suspensions at normal (20°C), low (4°C and −20°C) or high (40°C) temperatures. As the lag time was found to be directly proportional to the number of germinating conidia [19], the viability rate was estimated from the ratio between lag times before and after storage. Compared to storage at 20°C, increased lag times (7–10 h at cold and high temperatures, respectively) were recorded with the wild-type. Similar effects were observed for the *Δabdhn1* and *Δabdhn2* single mutant strains (**Fig. 9**). By contrast, storage at low (4°C), freezing (−20°C) or high (40°C) temperatures strongly decreased the germination capacity of *Δabdhn3* conidia. In the two latter conditions, germination of *ΔΔabdhn1-abdhn2* conidia was also affected.

### Impact of Dehydrin-like Proteins on *A. Brassicicola* Virulence

**Figure 10:**
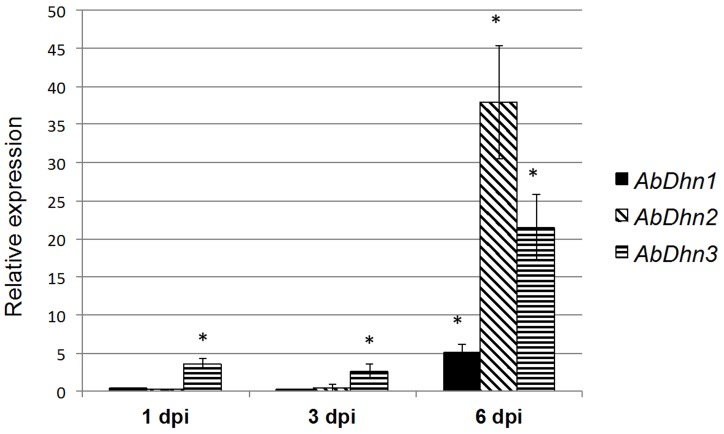
Quantitative RT-PCR results for the expression of *AbDhn* genes during the infection kinetics of *A. brassicicola* wild-type strain on *B. oleracea*. For each gene, expression induction is represented as a ratio of its relative expression at 1, 3 and 6(studied gene transcript abundance/tubulin transcript abundance) in each inoculated sample to its relative expression in free-living fungal control cultures. The experiment was performed twice on biologically independent samples with three technical replicates. Error bars indicate standard deviations and asterisks indicate a relative expression significantly different from 1 (Student test, *P*<0.01).

The expression of *AbDhn* genes was examined during infection of *Brassica oleracea* leaves inoculated with the wild-type strain (**Fig. 10**). Up to 3 days post-inoculation (dpi), no significant difference was noted between the *AbDhn* transcript levels in free-living mycelium (control) and those in infected leaves, while small necrotic symptoms were already observed. At 6 dpi, higher relative levels of *AbDhn* (in particular *AbDhn2* and *AbDhn3*) transcripts were recorded. At this stage, large typical necrotic areas with emerging conidia were apparent.

**Figure 11:**
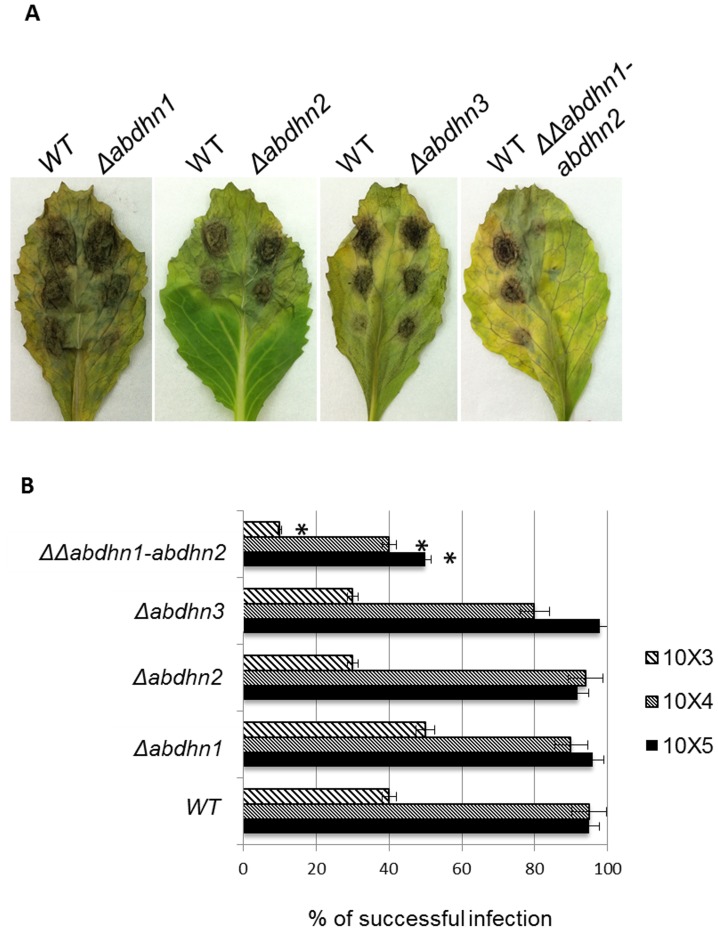
Effects of *AbDhn* knockouts on pathogenicity. *B. oleracea* leaves were inoculated with 5 µL drops of conidia suspension (10^5^, 10^4^ or 10^3^ conidia mL^−1^ in water). Mutants were inoculated on the right part of the central vein and compared on the same leaf with the parental strain (inoculated on the left part of the central vein). A: Representative result at 5 dpi. B: Percentage of successful infection at 5 dpi. The experiment was repeated twice and for each experiment each genotype was inoculated on 30 leaves at the three inocula concentrations. Error bars indicate standard deviations and asterisks indicate a significant difference with respect to the wild-type aggressiveness using the Student test (*P*<0.01).

The pathogenic behaviour of the dehydrin-like deficient-mutants was examined at both vegetative (leaves) and reproductive (siliques) plant developmental stages. When inoculated on intact *B. oleracea* leaves at decreasing inoculum concentrations (from 10^5^ to 10^3^ conidia per mL), irrespective of the inoculum pressure, no difference was recorded when the single deletion mutants were compared to the wild-type strain concerning the symptom aspect (**Fig. 11A**) or the rate of successful infection (**Fig. 11B**), estimated as the lesion size and percentage of typical lesions formed at 5 dpi, respectively. By contrast, the double deletion mutant was highly compromised in its pathogenicity, i.e. only small necrotic lesions with very limited spread around the inoculation sites were observed at 5 dpi on tissues inoculated with this strain.

**Figure 12:**
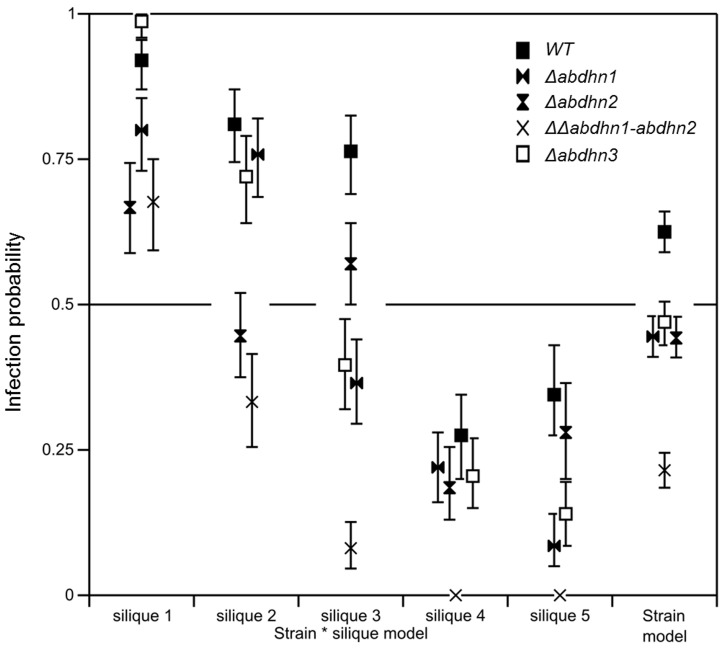
Transmission capacity of *A. brassicicola* wild-type (WT) and AbDhn-deficient genotypes to *Arabidopsis thaliana* seeds (L*er* ecotype). The seed transmission capacity according to the silique stage and global seed transmission capacity (strain model) were measured as described by Pochon et al [26]. The five youngest siliques of at least five plants were inoculated with each fungal genotype and the experiment was repeated twice. Contaminated siliques were harvested 10 dpi. After dissection, seeds were incubated separately on PDA medium for 2 days. A seed was considered contaminated when incubation resulted in typical *A. brassicicola* colony development. For each inoculated fungal genotype, the seed infection probability was evaluated from at least 1000 seeds. Values represent infection probabilities with 95% confidence interval.

When inoculated on siliques of *A. thaliana Ler* ecotype and recording seed transmission at 10 dpi, a slight but significant (*p*<0.05) decrease in global transmission rates was observed for the single *AbDhn* deletion mutants compared to the wild-type strain *Abra 43* (**Fig. 12** **)**. Much lower infection probabilities were obtained after inoculation with the ΔΔ*abdhn1-abdhn2* strain. This difference was statistically significant overall and for each silique (*p*<0.05). The gradient of fungal incidence from the oldest silique (n° 1) and the youngest one (n° 5) was observed for all tested genotypes.

## Discussion

Three proteins harbouring the fungal dehydrin signature pattern were identified from the *A. brassicicola* genome. *In silico* analyses confirmed that they shared the characteristic features of dehydrin-like proteins via their physicochemical properties. In this respect, *A. brassicicola* is similar to *A. fumigatus*, whose genome also encodes three different dehydrin-like proteins. Conversely, *DlpA* represents the only gene in the *A. nidulans* genome with sequence similarities to putative dehydrin proteins [12]. The pairwise identity rates indicated marked divergence between AbDhn1, AbDhn2 and AbDhn3 sequences, with less of 16% identity. They were not close to *Aspergillus* dehydrin-like proteins, with only a maximum of 27% identity between DlpA/AbDhn2 or DprB/AbDhn2. AbDhn3 differed substantially from other known dehydrins because of its large size and its specific expression pattern. This protein was indeed only weakly expressed in all conditions tested, except during plant tissue colonization. This surprising result suggested that AbDhn3 was specifically involved in a mechanism related to the fungal development in host plant, more particularly during the necrosis formation. *Abdhn3* expression might be induced following exposure to specific host metabolites, which differ from those (camalexin, brassinin, AlITC) used for *in vitro* expression analyses. Two AbDhn2 protein isoforms seemed to be produced by alternative splicing of *AbDhn2* pre-mRNA. We were unable to demonstrate particular functional characteristics of each isoform or specific accumulation under the conditions tested (data not shown).

Expression analyses suggested the involvement of AbDhn1/2 in the cellular response to oxidative, osmotic and cold stresses. They were also highly expressed in germinated conidia exposed to the brassicaceous defense metabolites, camalexin, brassinin and ITCs. Since the Hog1 MAPK cascade plays an important role in the adaptive response to phytoalexin exposure in *A. brassisicola* [16] and to oxidative and osmotic stress in several fungal species [20], [21], [22], we analysed AbDhn gene regulation in a mutant strain lacking the Hog1 MAP kinase. Q-PCR analysis of *AbDhn* expression revealed the absence of NaCl-induced *AbDhn* expression in the hog1 deletion strain, indicating that the Hog1 MAP kinase is involved in the regulation of *A. brassicicola* dehydrin genes, as previously reported for *Aspergillus* dehydrins [11], [12], [13].

Conversely, AbDhn proteins do not act downstream of AbHacA, the major UPR transcription regulator in *A. brassicicola* [17]. In agreement with our results, previous findings in *A. nidulans* and *A. fumigatus* showed that treatment with UPR inducers (dithiothreitol (DTT), tunicamycin, farnesol) triggered significant upregulation of dehydrins, suggesting their potential role as molecular chaperones to prevent the aggregation of proteins partially unfolded due to stress damage [11], [12], [13]. However, no obvious defect was seen in the growth of AbDhn single mutants upon supplementation with DTT (data not shown). We monitored basal expression of the three *A. brassicicola* dehydrin-like genes in a *AbHacA* mutant defective for the unfolded protein response. In the absence of exogenous stress, a dramatic increase in *AbDhn1* (and, to a lesser extent, of *Abdhn2* and *AbDhn3*) transcripts was observed in the Δ*abhacA* strain, compared to the wild-type. It was reported that a basal UPR exists in the absence of stress [23], [24]. This process should allow the cells to make minor adjustments necessary to buffer dynamic fluctuations in endoplasmic reticulum stress during hyphal growth and maintain continuous ER homeostasis. In a strain lacking the HacA UPR regulator, the basal maintenance of ER is difficult or impossible, which seems to cause cellular stress quite similar to that generated by chemical agents like DTT and tunicamycin. As HacA-targeted genes could not be induced in a cellular Δ*hacA* background, we hypothesized that other stress protective molecules, in particular dehydrins acting as chaperones, could be induced in a HacA-independent manner as a compensatory mechanism.

Although *AbDhn* gene expression is induced under various stresses, the knock-out approach showed that these dehydrin-like proteins had an impact mainly on oxidative stress tolerance. The corresponding deficient single mutants were indeed characterized by a strong susceptibility towards oxidative stress generated by exposure to menadione, H_2_O_2_ or Al-ITC. We previously showed that glucosinolate-derived isothiocyanates induced intracellular ROS accumulation in fungal cells [15]. These results are in good agreement with data from *Aspergillus* strains, indicating a role of DlpA and DprA in stress resistance against ROS [11], [12]. Fluorescent protein fusions suggested that AbDhn1 and AbDhn2 were associated with peroxisomes. These organelles are known to participate not only in ROS generation but also in cell rescue from the damaging effects of such radicals [25]. DprA and DprB were previously found to be important for peroxisome function and DprA mutants had altered catalase activity [11]. Catalases have a protective role against H_2_O_2_ and are localized in peroxisomes. The association of AbDhn1 and AbDhn2 with the peroxisomes is consistent with a possible perturbation of protective mechanisms to counteract oxidative stress and maintain the redox balance in AbDhn mutants.

Wong Sak Hoi et al [11] reported that DprB was involved in the response to osmotic stress. Although *AbDhn* expression seemed to be Hog1-dependent, all of the *A. brassicicola* dehydrin-like deficient mutants had normal tolerance to osmotic stress. In line with this observation, we previously reported that Δ*abhog1* mutants, while being hypersensitive to oxidative stress, had normal growth in the presence of high sorbitol concentrations [16], suggesting a minor role of this MAP kinase in the response of *A. brassicicola* to high osmolarity.

We also showed that storage at freezing (−20°C) or high (40°C) temperatures decreased the germination capacity of *Δabdhn3* conidia. Similarly, it has previously been reported that DrpC in *A. fumigatus* and DlpA in *A. nidulans* confer cold and heat tolerance of conidia, respectively [11], [12]. We also observed that high temperatures strongly affected germination of *ΔΔabdhn1-abdhn2* conidia suggesting a (partial) functional redundancy associated with the heat tolerance of these two proteins. In fact, our study highlighted other functional redundancies of the two proteins, which had very similar expression patterns, in developmental processes and during pathogenesis.

Concerning development, we observed that *ΔΔabdhn1-abdhn2* mutants only weakly sporulated. First dehydrin-encoding genes were identified in *T. borchii* and *A. fumigatus* during searches for genes controlling fruiting body maturation or conidial dormancy, respectively [6], [11]. In *A. fumigatus*, dehydrin transcipts were detected in dormant conidia, and then their levels decreased dramatically upon germination suggesting an important role for these proteins during conidial dormancy. This is also supported in *A. brassicicola* by the observation that conidia producing an AbDhn1- or AbDhn2-GFP fusion protein under control of their native promoters displayed strong fluorescence. However, this inability of dehydrin-deficient fungi to form conidia has not been previously reported in any fungal dehydrin single mutant and we showed for the first time that dehydrins were required for asexual sporulation.

Concerning pathogenesis, virulence of the wild-type and *AbDhn* mutants were first compared on *B. oleracea* host plants by leaf inoculation experiments. Secondly, we compared the abilities of mutants and the wild-type to transmit to *A. thaliana* seeds by using the model pathosystem recently described by Pochon et al [26]. In both cases, the double mutant *ΔΔabdhn1-abdhn2* was severely compromised in its pathogenicity. One previously reported function attributed to dehydrin is the protection against oxidative stress, which may be generated by the host plant defense system. Oxidative burst is a general plant defense mechanism that occurs at a very early stage of the interaction and is characterized by rapid accumulation of hydrogen peroxide in the extracellular space of plant tissues exposed to biotic stress [27], [28]. This oxidative burst occurs in many plant–pathogen interactions and leads to hypersensitive cell death (HR), which is thought to confine the pathogens to initial infection site. HR is efficient against biotrophic pathogens but it does not generally protect plants against infection by necrotrophic pathogens, such as *A. brassicicola*, which can utilize dead tissues [29]. However, necrotrophic lifestyle requires the induction of fungal detoxification mechanisms to overcome the toxic effects of reactive oxygen species (ROS). Mannitol was proposed to act as an antioxidant agent and protect *A. brassicicola* cells by quenching ROS [30]. Similarly, dehydrins may contribute to provide protection against oxidative stress. The decreased aggressiveness on *B. oleracea* and the lower capacity to be transmitted to *Arabidopsis* seeds via siliques observed for *ΔΔabdhn1-abdhn2* could therefore be related to their increased susceptibility to oxidative burst during the early leaf or silique infection stage. At a later infection stage, i.e. during leaf or silique tissue colonization, *A. brassicicola* is also exposed to glucosinolate-derived isothiocyanates that induce intracellular ROS accumulation in fungal cells [15]. *In planta* assays were conducted on leaves of *Brassica olerace*a var. Bartolo and fruits of *A. thaliana* ecotype L*er* that both accumulated various glucosinolates. In addition to their increased susceptibility to extracellular ROS, the low aggressiveness and seed colonization capacity of *ΔΔabdhn1-abdhn2* strain may thus also be related to their failure to overcome the intracellular oxidative stress caused by ITC during leaf or silique colonization. Surprisingly, while *AbDhn1* was found to be strongly expressed in *A. brassicicola* when exposed to various *in vitro* stresses (**Fig. 4**), in particular when exposed to host defence metabolites, this gene was only weakly up-regulated during leaf infection. However, its maximal expression could occur at earlier times than those that we analyzed (i.e. before 1 dpi). Indeed, it is likely that oxidative burst and production of phytoalexins occur in first hours of infection. The fact that the *AbDhn2* mutant did not exhibit a lower agressiveness may also seem surprising since its *in planta* expression is strongly induced. This could be explained by the existence of a functional redundancy between dehydrins, which would be partly suppressed in the double mutant.

In conclusion, these results highlight the importance of dehydrin proteins with respect to the ability of *A. brassicicola* to efficiently accomplish key steps of its pathogen life cycle. During tissue colonization, they probably participate in fungal protection against oxidative stress and are crucial factors in the vertical transmission mechanism (i.e. seed transmission). Moreover, AbDhn1 and AbDhn2 are required for asexual sporulation, which is necessary for efficient horizontal transmission of the pathogen, which is a key element of the fungal disease spreading process.

## Materials and Methods

### Strains and Growth Conditions

The *A. brassicicola* wild-type strain Abra43 used in this study has previously been described and used in the laboratory to generate various deletion mutants [31]. The Δ*abhog1* and Δ*abhacA* mutant strains deficient for the MAP kinase AbHog1 and the transcription factor AbHacA, respectively, have been formerly described [16], [17]. For routine culture, *A. brassicicola* was grown and maintained on potato dextrose agar (PDA, Biokar) supplemented with hygromycin B for mutants. To study hyphal growth in liquid media, conidial suspensions (10^5^ spores.ml^−1^, final concentration) were inoculated onto microplate wells containing the appropriate test substances in potato dextrose broth (Difco) in a total volume of 300 µl. Microplates were placed in a laser-based microplate nephelometer (NEPHELOstar, BMG Labtech) and growth was monitored automatically over a 33 h period. Nephelometry, which is based on the measurements of scattered light, was proved to be an accurate indicator of the fungal biomass and can be used as reliable tool for the monitoring of fungal growth [19]. Data were exported from Nephelostar Galaxy software in ASCII format and further processed in Microsoft Excel. Two variables, i.e. lag time and maximal slope, were calculated from the growth curves using the calculation method reported by Joubert et al [19]. At least three replicates per treatment were used. To study the susceptibility of the fungal strains to ITC, allyl-ITC (AlITC), purchased from Aldrich Chemical Co. (Milwaukee, WI), was diluted from stock solutions prepared in methanol at the final desired concentrations. The phytoalexin camalexin was synthesized according to Ayer et al [32] and brassinin according to Kutschy et al [33] and Takasugi et al [34]. Stock solutions were prepared in DMSO and added to the medium at the desired concentrations. Solvent concentrations in controls and assays did not exceed 1% (v/v).

### In Silico Analysis

Blast analyses against the *A. brassicicola* genome and other fungal genomes were submitted to the Joint Genome Institute (http://genomeportal.jgi-psf.org/Altbr1/Altbr1.home.html) and National Center for Biotechnology Information (http://www.ncbi.nlm.nih.gov/sutils/genom_table.cgi?organism=fungi). Estimation of intrinsic protein disorder and prediction of protein binding regions in disordered proteins were performed online using different web servers: IUPred and ANCHOR (http://anchor.enzim.hu/, [35]) and PrDOS (http://prdos.hgc.jp/cgi-bin/top.cgi, [36] ). Protein hydrophilicity analyses were performed based on the Kyte and Doolittle algorithm [37] by calculating their Grand Average of Hydropathy (GRAVY) index (http://www.gravy-calculator.de).

### RNA Isolation and Expression Analysis by Real-time Quantitative PCR

Total RNA was prepared according to the TRIzol reagent protocol (Invitrogen). Additional cleanup and DNase treatment were performed using the Nucleospin RNA II kit (Macherey-Nagel) according to the manufacturer’s protocol. First-strand complementary DNA was synthesized from 5 µg of total RNA and used for real-time PCR. Amplification experiments were conducted as previously described [17]. The relative quantification analysis was performed using the comparative ΔΔCt method as described by Winer et al [38]. To evaluate the gene expression level, the results were normalized using Ct values obtained from *β-*tubulin cDNA amplifications run on the same plate.

### DNA Procedure and Southern Hybridization

Genomic DNA was extracted from mycelium according to Moller et al [39]. For Southern analysis, DNA fragments resulting from genomic DNA digestion with *Pst*I or *Sac*I were separated on 1% agarose gels and vacuum transferred to Hybond N membranes (Amersham Biosciences). Blots were then probed with a PCR product that was amplified from *A. brassicicola* genomic DNA and ^32^P labelled using the Random Prime Labelling System Rediprime II (Amersham Biosciences).

### Generation of Targeted Gene Knockout Mutants and Fusion Strains

The gene replacement cassettes were generated using the double-joint PCR procedure described by Yu et al [40]. The selectable marker inserted in the PCR constructs corresponded to the *Hph* gene cassette (1436 bp) from pCB1636 [41] or the *Nat* gene cassette (2150 bp) from pNR2 [42] conferring resistance to hygromycinB and nourseotricin, respectively. Two sets of primers (Table S1) were used with Phusion Hot Start High-Fidelity DNA Polymerase (Finnzymes, Espoo, Finland) to amplify at least 500 bp from the 5′ and 3′ flanking regions of each targeted gene. The double-joint final PCR was purified and used to transform *A. brassicicola* protoplasts as described by Cho et al [43]. *A. brassicicola* wild-type Abra43 was used to obtain single hygromycin resistant transformant strains Δ*abdhn1–3*. Δ*abdhn1* genotype was used to obtain ΔΔ*abdhn1-abdhn2* hygromycin and nourseotricin resistant strains. The hygromycin resistant mutants were selected and prescreened by PCR with relevant primer combinations to confirm integration of the replacement cassette at the targeted locus. The gene replacement mutants were further purified by three rounds of single-spore isolation and then confirmed by Southern blot analysis. The *AbDhn* C-terminal GFP fusion constructs were generated by fusion PCR as described in **Fig. S5**. Using *A. brassicicola* genomic DNA as template, the respective ORFs and 3′ flanking regions were amplified with relevant primer combinations (**Table S1**). In parallel, fragments containing the sGFP and Hyg B cassettes were amplified from the plasmid pCT74 [18] and pCB1636, respectively. The resulting PCR fragments were mixed and subjected to second fusion PCR. A linker containing three glycine residues was introduced at the 3′ end of the respective ORFs to replace the stop codons. The final PCR products were transformed in the *A. brassicicola* wild-type (as described above) to make AbDhn–GFP fusion mutants. Transformants with expected genetic integration events were identified by PCR. Labelling peroxisomes in strains expressing AbDhn-GFP fusion was performed by transformation with a linear *Nat–DsRed-SKL* cassette amplified from plasmid pDsRed-SKL [44]. Observations were performed under a Nikon (Nikon Instruments, Melville, NY) A1S1 confocal laser microscope equipped with argon-ion (488 nm) and diode (561 nm) lasers.

### Western Blot Analysis

Production of AbDhn proteins in *A. brassicicola* strains expressing C-terminal GFP fusion was monitored by Western blot using HRP coupled antibodies directed against GFP (Miltenyi Biotec, Germany). Samples were prepared from mycelia obtained by growing conidial suspensions at 25°C for 24 h in PDB (10^5^ conidia mL^−1^) and then exposed to 350 mM NaCl final concentration for 2 h. Mycelia were collected by filtration on filter paper, ground with a mortar and pestle to a fine powder under liquid nitrogen and homogeneized in ice-chilled buffer (500 mM Tris-HCl, pH 8.7, 700 mM sucrose, 50 mM EDTA, 100 mM KCl, 2% (v/v) β-mercaptoethanol, 1 mM PMSF, 50 mM NaF, 5 mM Na pyrophosphate, 0.1 mM Na vanadate, 10 mM β-glycerophosphate). Proteins were then extracted with one volume of water-saturated phenol and precipitated from the phenolic phase with five volumes of 100 mM NH_4_^+^ acetate prepared in methanol. The protein concentration in the extracts was calculated using a BCA protein assay reagent (Pierce, Rockford, IL). Equal quantities (10 µg) of protein samples were loaded on 10% polyacrylamide gels and blotted onto nitrocellulose membranes (Schleicher and Schuell, Dassel, Germany). Antibody binding was visualized using an ECL Plus kit Western (Amersham Biosciences, Buckinghamshire, UK).

### Pathogenicity Assays

For plant infection assays on *Brassica oleracea* plants (var. Bartolo), 5 µL drops of *A. brassicicola* conidia suspension (10^5^,10^4^ or 10^3^ conidia mL^−1^ in water) were inoculated on leaves from 5 week-old plants. Inocula were symmetrically deposited on the left and right sides from the central vein. The plants were then maintained under saturating humidity (100% relative humidity). The experiment was repeated twice and for each experiment each genotype was inoculated onto 30 leaves at the three inoculum concentrations.

Seed contamination assessments were estimated as described by Pochon et al [26]. Two 2.5 µL drops of an *A. brassicicola* conidial suspension (1×10^5^ conidia mL^−1^ in water) supplemented with 0.01% (v/v) Tween 20 were placed on the five youngest siliques (one drop at the silique base and one in the middle) from 1-month-old *A. thaliana* L*er* plants. At least five plants per fungal genotype were inoculated and the experiment was repeated twice. As a control for all experiments, two 2.5 µL drops of a 0.01% (v/v) Tween 20 solution were placed on five siliques of one plant. The plants were then maintained under saturating humidity for 2 days in the dark. Contaminated siliques were harvested 10 days after inoculation. Inoculated or control siliques were dissected with sterile forceps and seeds were carefully harvested to avoid contact with the fungus potentially present on the outer surface of siliques. Seeds were incubated separately on PDA medium for 2 days. A seed was considered contaminated when incubation resulted in typical *A. brassicicola* colony development. Seed contamination data were analyzed using logistic (logit) generalized linear models as previously described [26].

## Supporting Information

Figure S1 **Verification of deletion mutants.** A : Schematic representation of the *AbDhn1, AbDhn2* and *AbDhn3* loci (light-gray boxes) with flanking regions (dark-gray boxes) and replacement constructs with the hygromycin B (*Hph*) and nourseotricin (*Nat*) resistance genes (white boxes). The positions of *Sac*I and *Pst*I sites, probes and the sizes of expected hydridizing fragments are shown. B: Southern hybridization of genomic DNA from wild-type Abra43 (WT) and transformants. Each DNA was digested with either *Sac*I or *Pst*I and the blot hybridized with the *Abdhn1*, *Abdhn2*, or *Abdhn3* probes.
(TIF)

Figure S2 **AbDHN2 amino acid sequence.** The eleven amino acids repeat at the N-terminal end of the sequence is indicated in red and blue characters. The five conserved DPR motifs are indicated in bold characters. Residues in italic are absent in the ß isoform and the underlined residue corresponds to a potential phosphorylated serine present only in the α isoform.
(TIF)

Figure S3 **Constitutive GFP expression in an** ***A. brassicicola*** **mutant.** Observation was performed using confocal microscopy. Bars = 10 µm.
(TIF)

Figure S4 **Comparison of growth rates of the wild-type strain and dehydrin-like deficient mutants in solid and liquid nutritive media.** A: *In vitro* growth tests were carried out on PDA plates. Growth was recorded after 7 days of incubation at 24°C. B: Nephelometric monitoring of the growth of different genotypes. Conidia from the wild-type and mutants were used to inoculate microplate wells containing standard PDB medium. Growth was automatically recorded for 25 h at 25°C using a nephelometric reader. The unit of the Y-axis corresponds to the Relative Nephelometric Unit (RNU). Lag time and maximal growth rate variables were calculated from the growth curves using a calculation method described by Joubert et al [19]. For each parameter, Student’s T-test was used to assess significant difference between mutants and parental isolate.
(TIF)

Figure S5 **Schematic representation of the construction of AbDhn-Gfp fusion cassette.** Targeted ORF and 3′ flanking region were amplified from *A. brassicicola* wild-type genomic DNA with two sets of primers (P1/P2 and P3/P4). Reverse primer P2 was designed so that the original stop codon was replaced by three Glycin codons. The *sGfp* coding sequence (stripped boxes) and *Hph* gene cassette were amplified from pCT74 [18] and pCB1636 [41], respectively. The four PCR fragments were fused using the double-joint PCR procedure described by Yu et al [40]. The resulting DNA fragment was introduced into *A. brassicicola* by protoplasts transformation and double crossing–over events replaced the wild locus by the *AbDhn-sGfp-Hph* cassette. Table S1 lists the different primers that were used for each *AdDhn* gene: P1 = GfpDHN-F1, P2 = GfpDHN-R1, P3 = GfpDHN-F2, P4 = GfpDHN-R2, P5 = Gfp-F, P6 = Gfp-R, P7 = M13F, P8 = M13R.
(TIF)

Table S1 **List of primers used in this study.**
(DOCX)
