# Supplementary figures and images for: Dehydrin-like Proteins in the Necrotrophic Fungus Alternaria brassicicola Have a Role in Plant Pathogenesis and Stress Response
Source: PLoS One. 2013 Oct 2;8(10):e75143. doi: 10.1371/journal.pone.0075143 (PMC3788798; doi:10.1371/journal.pone.0075143)

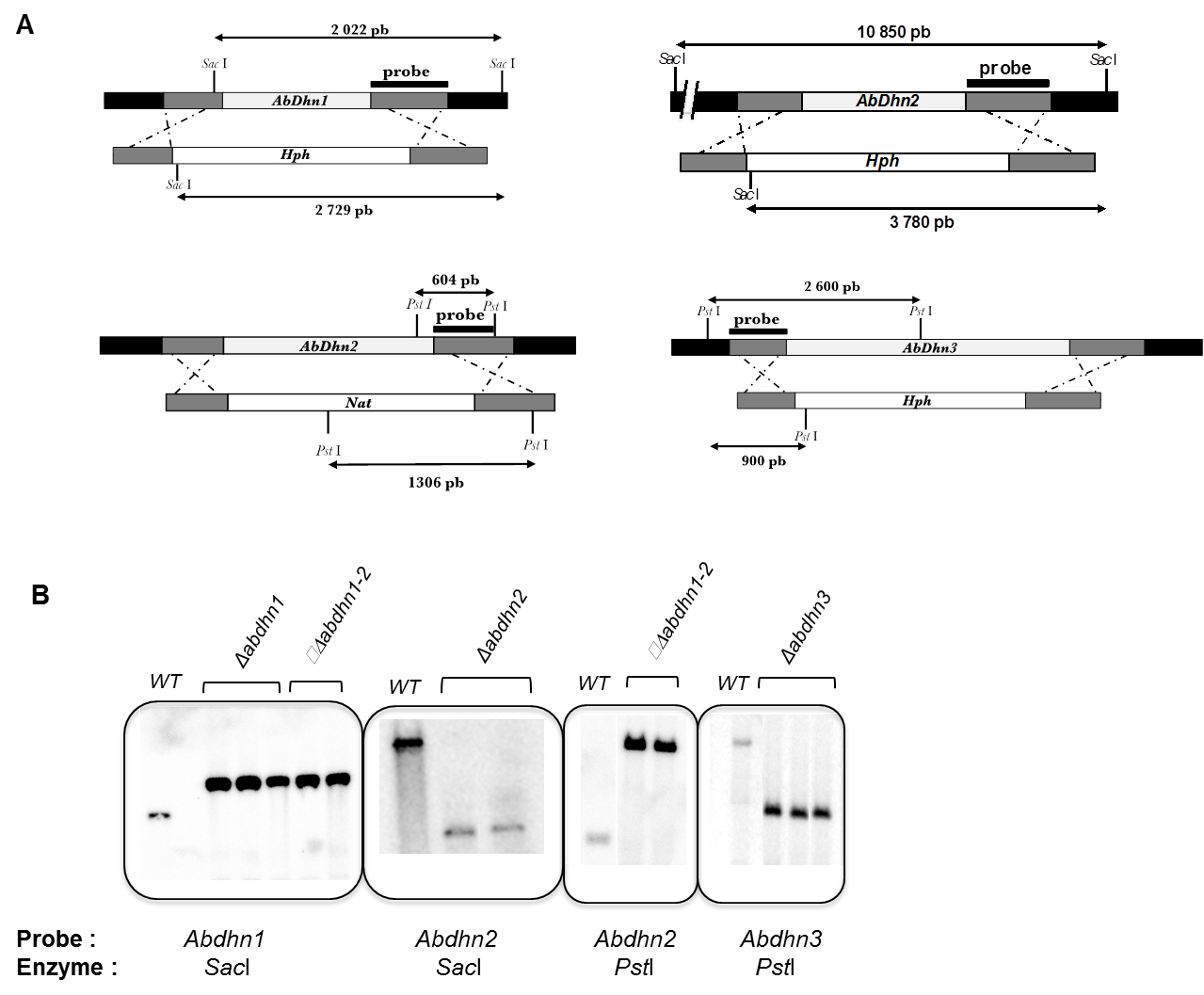

Supplement: Figure S1 — Verification of deletion mutants. A : Schematic representation of the AbDhn1, AbDhn2 and AbDhn3 loci (light-gray boxes) with flanking regions (dark-gray boxes) and replacement constructs with the hygromycin B (Hph) and nourseotricin (Nat) resistance genes (white boxes). The positions of SacI and PstI sites, probes and the sizes of expected hydridizing fragments are shown. B: Southern hybridization of genomic DNA from wild-type Abra43 (WT) and transformants. Each DNA was digested with either SacI or PstI and the blot hybridized with the Abdhn1, Abdhn2, or Abdhn3 probes. (TIF) [file pone.0075143.s001.tif]

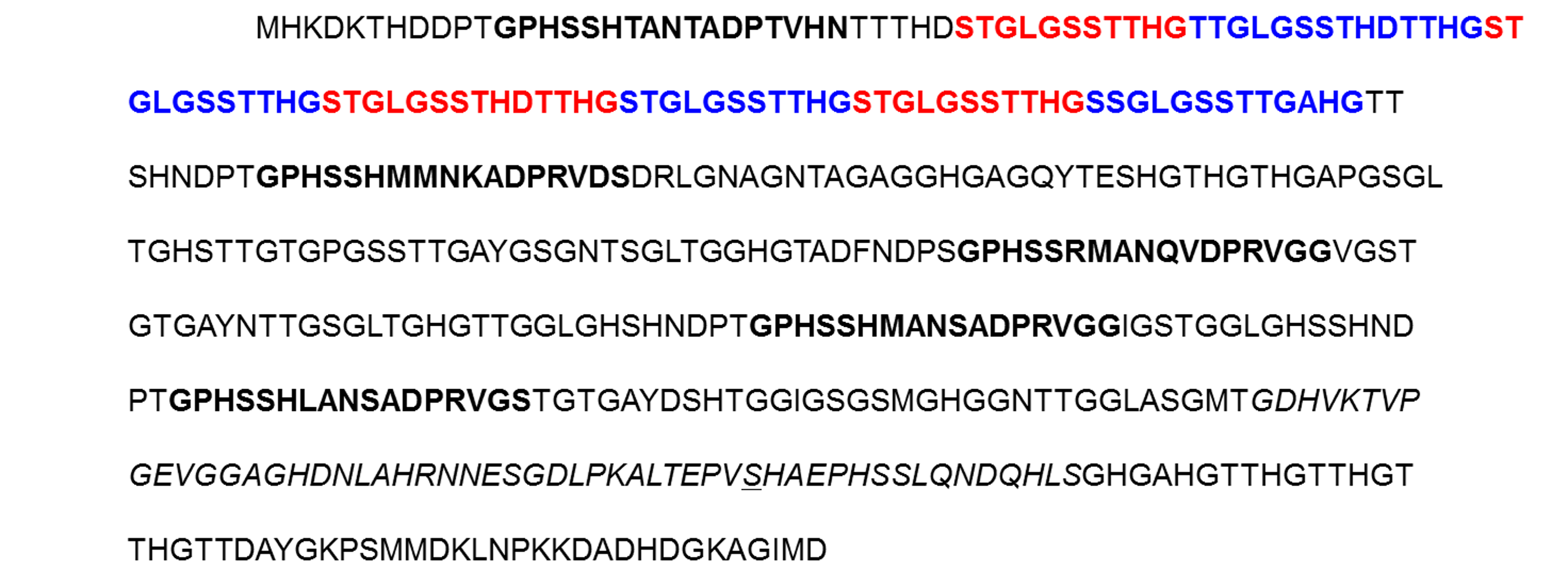

Supplement: Figure S2 — AbDHN2 amino acid sequence. The eleven amino acids repeat at the N-terminal end of the sequence is indicated in red and blue characters. The five conserved DPR motifs are indicated in bold characters. Residues in italic are absent in the ß isoform and the underlined residue corresponds to a potential phosphorylated serine present only in the α isoform. (TIF) [file pone.0075143.s002.tif]

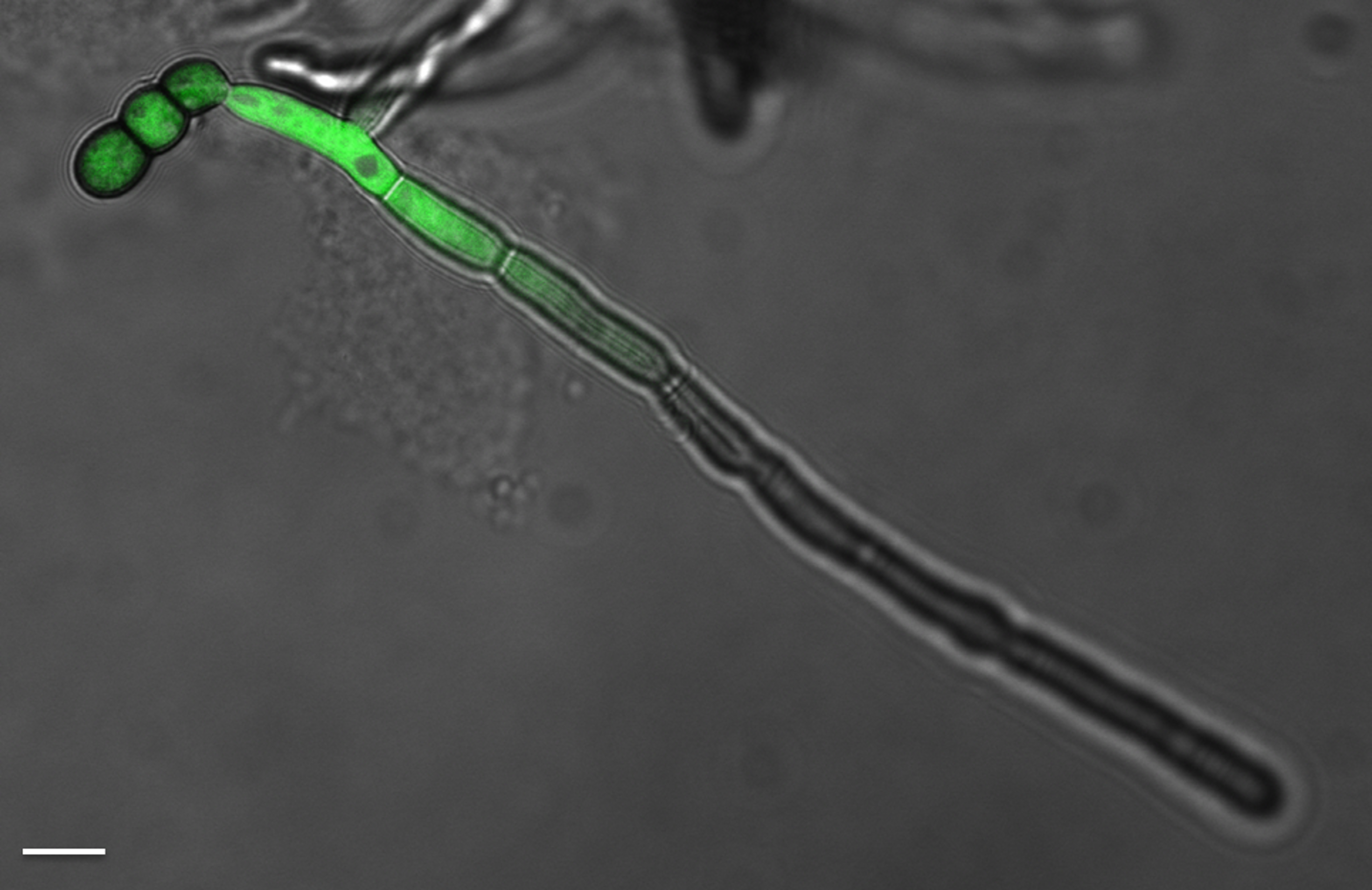

Supplement: Figure S3 — Constitutive GFP expression in an A. brassicicola mutant. Observation was performed using confocal microscopy. Bars = 10 µm. (TIF) [file pone.0075143.s003.tif]

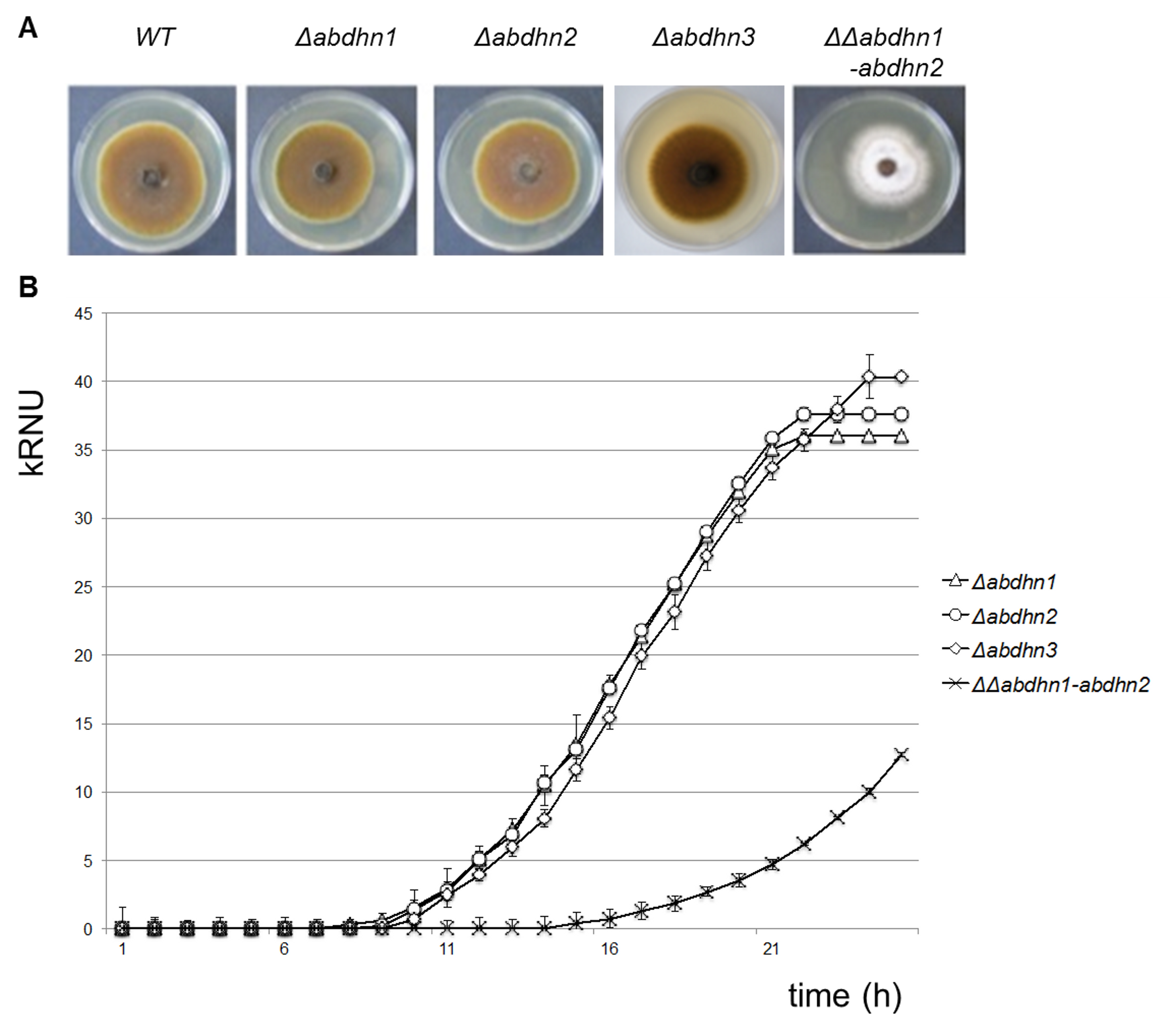

Supplement: Figure S4 — Comparison of growth rates of the wild-type strain and dehydrin-like deficient mutants in solid and liquid nutritive media. A: In vitro growth tests were carried out on PDA plates. Growth was recorded after 7 days of incubation at 24°C. B: Nephelometric monitoring of the growth of different genotypes. Conidia from the wild-type and mutants were used to inoculate microplate wells containing standard PDB medium. Growth was automatically recorded for 25 h at 25°C using a nephelometric reader. The unit of the Y-axis corresponds to the Relative Nephelometric Unit (RNU). Lag time and maximal growth rate variables were calculated from the growth curves using a calculation method described by Joubert et al [19]. For each parameter, Student’s T-test was used to assess significant difference between mutants and parental isolate. (TIF) [file pone.0075143.s004.tif]
