# Supplementary material for: Dehydrin-like Proteins in the Necrotrophic Fungus Alternaria brassicicola Have a Role in Plant Pathogenesis and Stress Response
Source: PLoS One. 2013 Oct 2;8(10):e75143. doi: 10.1371/journal.pone.0075143 (PMC3788798; doi:10.1371/journal.pone.0075143)
Supplement: Table S1 — List of primers used in this study. (DOCX) [file pone.0075143.s006.docx]

**Table S1:** List of primers used in this study

| **Name** | | **Sequence** | **Description** | |
| --- | --- | --- | --- | --- |
| FusDHN1-F1  FusDHN2-F1  FusDHN3-F1 | | ^5’^ GGACTGAAATGCGAATTGGT ^3’^  ^5’^ TGCTATCCGGGTACTCTTGC ^3’^  ^5’^ CAGCTGCCCTTTCTATTGGA ^3’^ | Set of primers used to generate, by double joint PCR, the AbDhn disruption cassette carrying the HygB resistance gene from pCB1636 | |
| FusDHN1-R1  FusDHN2-R1  FusDHN3-R1 | | ^5’^*GTCGTGACTGGGAAAACCCTGGCG*CGCGCGTAGAGGCTGGAGTATC ^3’^  ^5’^*GTCGTGACTGGGAAAACCCTGGCG*TCGGCCTTGTTCATCATGT ^3’^  ^5’^*GTCGTGACTGGGAAAACCCTGGCG*CCTGTGCTCATCTGGTCGTA ^3’^ |  |  |
| FusDHN1-F2  FusDHN2-F2  FusDHN3-F2 | | ^5’^*TCCTGTGTGAAATTGTTATCCGCT*CGTACCAACCGCCTCTACTC ^3’^  ^5^’*TCCTGTGTGAAATTGTTATCCGCT*CAATGAGAGTCGGCTTGTGA^3’^  ^5^’*TCCTGTGTGAAATTGTTATCCGCT*ACAGGAATGCTGCTCTTGGT^3’^ |  |  |
| FusDHN1-R2  FusDHN2-R2  FusDHN3-R2 | | ^5’^ GGCTCGCTATTCATCGTCTT ^3’^  ^5’^ CACTTCTGGGCCACCATATC ^3’^  ^5’^ ACGATGCTCGTCTTTCTCGT ^3’^ |  |  |
| FusDHN1-NF  FusDHN2-NF  FusDHN3-NF | | ^5’^ GAGGCTGGAGGTGGTAATCA ^3’^  ^5’^ ACTAGGCCAATGTCGACCAG ^3’^  ^5’^ CTACAACAAACGCCATCGTG ^3’^ |  |  |
| FusDHN1-NR  FusDHN2-NR  FusDHN3-NR | | ^5’^ AACTCATCGTCCGAGTCAGG ^3’^  ^5’^ GCTACGTACGCACACAAGGA ^3’^  ^5’^ GTTGCTCCTTCTCCTTGTCG ^3’^ |  |  |
| GfpDHN1-F1  GfpDHN2-F1  GfpDHN3-F1 | | ^5’^GAGCATGACGTCACACCTTC ^3’^  ^5’^ GGAGCTTACGGCTCAGGCAA ^3’^  ^5’^ TCCAACCGAAACGATACTCC ^3’^ | Set of primers used to generate, by double joint PCR, the AbDhn-Gfp fusion cassette carrying the GFP coding sequence from pCT74 and the HygB resistance gene from pCB1636 | |
| GfpDHN1-R1  GfpDHN2-R1  GfpDHN3-R1 | | ^5’^*CTCCTCGCCCTTGCTCACCAT*TCCTCCTCCTCTACGTGTCTGGTAGCCCGT ^3’^  ^5’^*CTCCTCGCCCTTGCTCACCAT*TCCTCCTCCGTCCATAATGCCAGCCTTGC ^3’^  ^5’^ *CTCCTCGCCCTTGCTCACCAT*TCCTCCTCCTATTGGGTCCTTGACGAGAC ^3’^ |  |  |
| GfpDHN1-F2  GfpDHN2-F2  GfpDHN3-F2 | | ^5^’*TCCTGTGTGAAATTGTTATCCGCT*ACGATGCACTGTGCACTTGG ^3’^  ^5^’*TCCTGTGTGAAATTGTTATCCGCT*ACGCTGTCTCCTCTTCTTGC ^3’^  ^5^’ *TCCTGTGTGAAATTGTTATCCGCT*CTACAACGACCCTGCTACTC ^3’^ |  |  |
| GfpDHN1-R2  GfpDHN2-R2  GfpDHN3-R2  Gfp-F  Gfp-R | | ^5’^ GCAATACTCATAAGCCCATG ^3’^  ^5’^ TTTGCCACATACTGGACCGA ^3’^  ^5’^ GCAGGAGACTTCTCTTTCTT ^3’^  ^5’^ GGAGGAGGAATGGTGAGCAAGGGCGAGGAG^3’^  ^5’^ *GTCGTGACTGGGAAAACCCTGGCG*CTAGAGGATCCCCTTGTACAGC^3’^ |  |  |
| Hph-F  Hph-R  Nat-F  Nat-R | | ^5’^ CGTTGCAAGACCTGCCTGAA ^3’^  ^5’^GGATGCCTCCGCTCGAAGTA ^3’^  ^5’^ TTCGGTTCCCTTTCTCCT ^3’^  ^5’^ACATCCACGGGACTTGAGAC ^3’^ | Forward and reverse *Hph* and *Nat* specific primers for transformant validation | |
| M13F | ^5’^ CGCCAGGGTTTTCCCAGTCACGAC ^3’^ | | Forward and reverse primers used to amplify the *Nat* cassette, the *Hph* cassette and the *Nat – DsredSKL* cassette from pNR2, pCB1636 and pDsRed-SKL, respectively |  |
| M13R | ^5’^ AGCGGATAACAATTTCACACAGGA ^3’^ | |  |  |
| OrfDHN2-F1 | | ^5’^ACTAGGCCAATGTCGACCAGCT^3’^ | Forward and reverse *AbDhn2* specific primers for amplification of full-length coding sequence | |
| OrfDHN2-R1 | | ^5’^ CTATCCATAATGCCAGCCTTGC^3’^ |  |  |
| ExpDHN1-F1  ExpDHN1-R1  ExpDHN2-F1  ExpDHN2-R1  ExpDHN3-F1  ExpDHN3-R1  ExpTUB-F1  ExpTUB-R1 | | ^5’^ CGTGGACCATCGTGCTGAT ^3’^  ^5’^ GCATCGTCTATCTACGTGTCTGGTA ^3’^  ^5’^ TCACATGATGAACAAGGCCG ^3’^  ^5’^ TTTCCAAGGCGGTCAGAATC ^3’^  ^5’^ TGCACCGTCTGAGGAACGA ^3’^  ^5’^ TGGCGAGTTTCGAGACCTTG ^3’^  ^5’^ TTCAACGAAGCCTCCAACAAC ^3’^  ^5’^ GTGCCGGGCTCGAGAT ^3’^ | Set of primers used for relative quantification of *AbDhn* genes expression by real-time PCR | |
